# Changing patterns in aerosol vertical distribution over South and East Asia

**DOI:** 10.1038/s41598-020-79361-4

**Published:** 2021-01-11

**Authors:** M. Venkat Ratnam, P. Prasad, S. T. Akhil Raj, M. Roja Raman, Ghouse Basha

**Affiliations:** 1 National Atmospheric Research Laboratory, Gadanki, 517112 India; 2 Research Centre for Environmental Changes, Academia Sinica, Taipei, 11529 Taiwan

**Keywords:** Atmospheric science, Climate change

## Abstract

Changing patterns in aerosol concentrations over the Asian region is well documented with a concurrent increase over India and a marked reduction over China. However, aerosol vertical distribution in the changing climate is not fully understood. By combining long-term satellite observations from MODIS and CALIOP, here we show rapid changes in the aerosol vertical distribution over the South and East Asia covering India and China. A statistically significant decreasing (increasing) trend in the boundary layer (free troposphere) aerosol concentrations is noticed over India. ERA-Interim reanalysis model suggests that this increase in free tropospheric aerosol concentrations are due to the lifting of boundary layer pollutants through an increase in convection (and vertical velocity) in a changing climate. In contrast, a consistent decreasing trend is observed over China irrespective of the altitude. Interestingly, a decreasing trend in Aerosol Optical Depth is observed over the northwest India and we relate this to an observed increase in precipitation leading to increase in the vegetation. It is also found that long-term oscillations like QBO, ENSO and solar cycle significantly affect the aerosol concentrations. Thus, it is prudent to conclude that background meteorology and dynamics play an important role in changing patterns of aerosol vertical distribution.

## Introduction

Atmospheric aerosols being emitted from different sources have a huge impact on climate, radiation budget and cloud microphysics through the scattering and absorption of incoming solar radiation in the Earth’s atmosphere^1–3^. Aerosol emissions are integrally linked to society through transport, industry, health, and other factors^4^. Thus, changes to the causes and effects of these emissions may alter the risks of a range of climate-related impacts. In recent decades, the optical properties of aerosols and their concentrations are the largest sources of uncertainty to assess the current global climate change^4^. It is well reported that the optical properties of aerosols and their distributions are greatly varying with space and time^5^. Extensive amounts of anthropogenic aerosols with complex chemical, physical and optical characteristics are being emitted in the South and East Asia^6^. Some recent studies using coupled climate model analysis for the last three decades brings out the effect of enhanced anthropogenic emissions on the Asian monsoon circulation and resultant precipitation over East and South East Asia^7,8^. However, a very recent analysis of long-term satellite measurements revealed a dipole pattern in Aerosol Optical Depth (AOD) with a marked reduction over China and a concurrent increase over India^5^. Several recent studies also reported a consistent decreasing trend in aerosol concentrations over China in the last decade due to ‘clean air actions’^9,10^, conversely over India and adjoining seas, a rapid increase in aerosol loading is observed^11^. In contrast, the decreasing trend in boundary layer aerosols over India is reported^12^. Using long-term ground-based observations, Ravi Kiran et al*.* noticed a decreasing trend in [Black Carbon (BC)] aerosol concentration over Gadanki (13.5° N, 79.2° E), India^12^. Similar features are reported in the surface concentrations using a network of observations over India, but interestingly, an increasing trend in the free tropospheric aerosol (BC) concentrations are reported^13^. The decreasing trend in AOD and PM2.5 over China in recent times is attributed to the meteorological parameters to a large extent besides emission^14,15^. Using measurements from 34 stations over China, a large decreasing trend in BC is also reported^16^. Earlier studies using ground-based and space borne observations along with coupled model simulations confirm a definite shift in the aerosol pattern (spatial and vertical) in the recent decade^7^, and thus it will be interesting to investigate the changes in the aerosol vertical distribution in changing climate over South and East Asian regions. Though, the long-term trends in surface aerosols and columnar integrated aerosol distribution over South and East Asia are well reported, the trends in aerosol vertical distribution are not fully understood.

The present study is oriented to investigate the long-term trends in aerosol vertical distribution over South and East Asia in the perspective of meteorology and dynamics of various parameters (boundary layer, surface temperature, vegetation, rainfall, convection, etc.,) on the changing aerosol patterns. This study uses both passive and active remote sensing satellites to obtain the spatial distribution of aerosol. Passive sensors like MODerate resolution Imaging Spectroradiometer (MODIS) on-board the Terra and Aqua satellites mainly provide the integrated aerosol property such as AOD with a good spatial and temporal resolution, however, knowledge on the vertical distribution is missing^17^. To bridge this gap, active remote sensing satellite like Cloud-Aerosol LIdar with Orthogonal Polarization (CALIOP) sensor on-board Cloud-Aerosol Lidar and Infrared Pathfinder Satellite Observations (CALIPSO) is used that provides the vertical distribution of aerosol physical and optical properties with reasonably good vertical and spatial resolution^18–20^. By combining these two independent satellites, vertical and spatial distribution of aerosol concentrations in a changing climate is investigated in the present communication. The observed climatic variations in the aerosols at different levels of the atmosphere are then linked to the changing patterns in meteorology and dynamics.

## Results

### Aerosol distribution over South and East Asia

**Figure 1:**
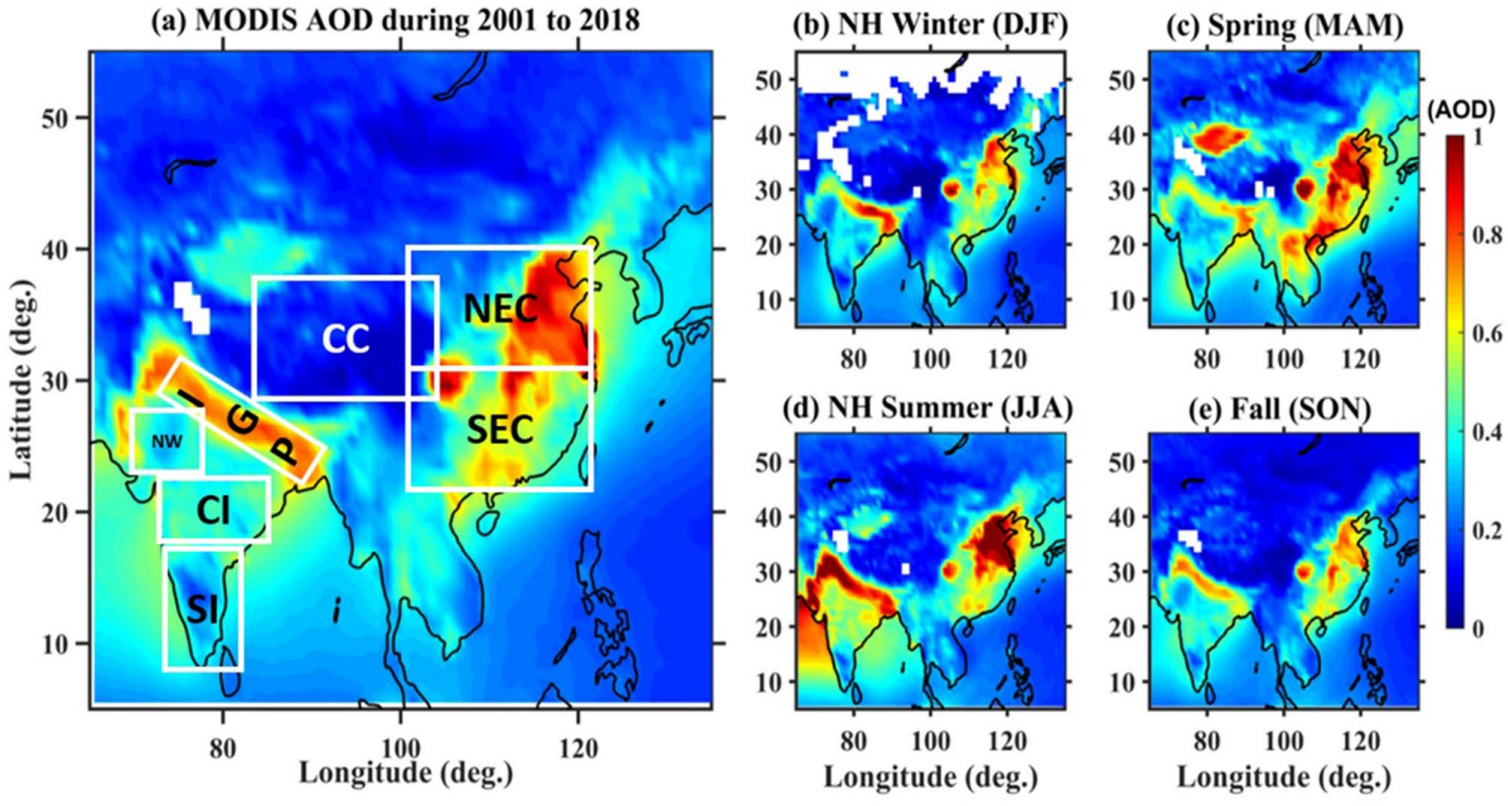
(**a**) Aerosol optical depth (AOD) climatology over South and East Asia observed using MODIS averaged during 2001–2018. Seasonal variation in AOD observed during (**b**) NH Winter (DJF), (**c**) Spring (MAM), (**d**) NH Summer (JJA) and (**e**) Fall (SON) seasons averaged during the same period. Different regions in (**a**) shown in the boxes represent south India (SI), central India (CI), northwest India (NW), Indo-Gangetic Plains (IGP), Central China (CC), northeast China (NEC) and southeast China (SEC). The maps were created by using MATLAB software R2019b with mapping tool box (see https://in.mathworks.com/products/new_products/release2019b.html and https://www.mathworks.com/products/mapping.html).

Climatological mean AOD over South and East Asia covering India and China obtained using MODIS averaged during 2001–2018 is shown in Fig. 1a. Large spatial variation in AOD is noticed over both India and China. High AODs (> 0.7) exists over Indo-Gangetic Plain (IGP) region and northeast China and very low AODs (< 0.2) prevails over south India and southwest China. Enhanced AOD over the northeast and central China (Hubei, Hunan and Henan provinces) is mainly due to industrialization as well as the most densely populated area^9^. Strong seasonal variation in the spatial pattern of AOD is noticed over both India and China. During the northern hemisphere (NH) winter (December–February) season (Fig. 1b), high AOD is seen along the IGP region mainly over eastern parts of India and northeast China. Relatively lower AOD over the south and central India and west China is noticed. This is mainly due to anthropogenic activity coupled with local emissions and meteorological conditions. In the spring (March–May) season (Fig. 1c), moderate AOD is observed over the entire Indian region but enhanced AOD over east China. During NH summer (June–August) season (Fig. 1d), maximum aerosol loading over the Arabian Sea and moderate over the central and northern landmass of India are seen. Whereas enhanced AOD is restricted to northeast China. Irrespective of the season, high AOD over the eastern part of China is attributed to the dense population (70%) living over that region which shows a clear relationship between the density of the population and the aerosol concentration^21^. In spite of the rainy season over India, where chances of wet scavenging (rainout or washout) are high, enhanced AODs can be noticed during this season. This is partly due to aerosol long-range transport through low-level jet and tropical easterly jet which persists over the Indian region and due to a greater number of break spells during monsoon season^22,23^. During the fall (September–November) season (Fig. 1e), large AOD is observed over northwest India due to crop residue burning emissions that are common in Punjab and Haryana^24^. These will be transported to the IGP regions and the eastern part of the Bay of Bengal (BoB). Interestingly, southern India and central China shows almost cleaner environment with very low AOD (< 0.2) in all seasons when compared to other parts. It will be interesting to see the long-term trends in these patterns.

### Long-term trends in AODs

**Figure 2:**
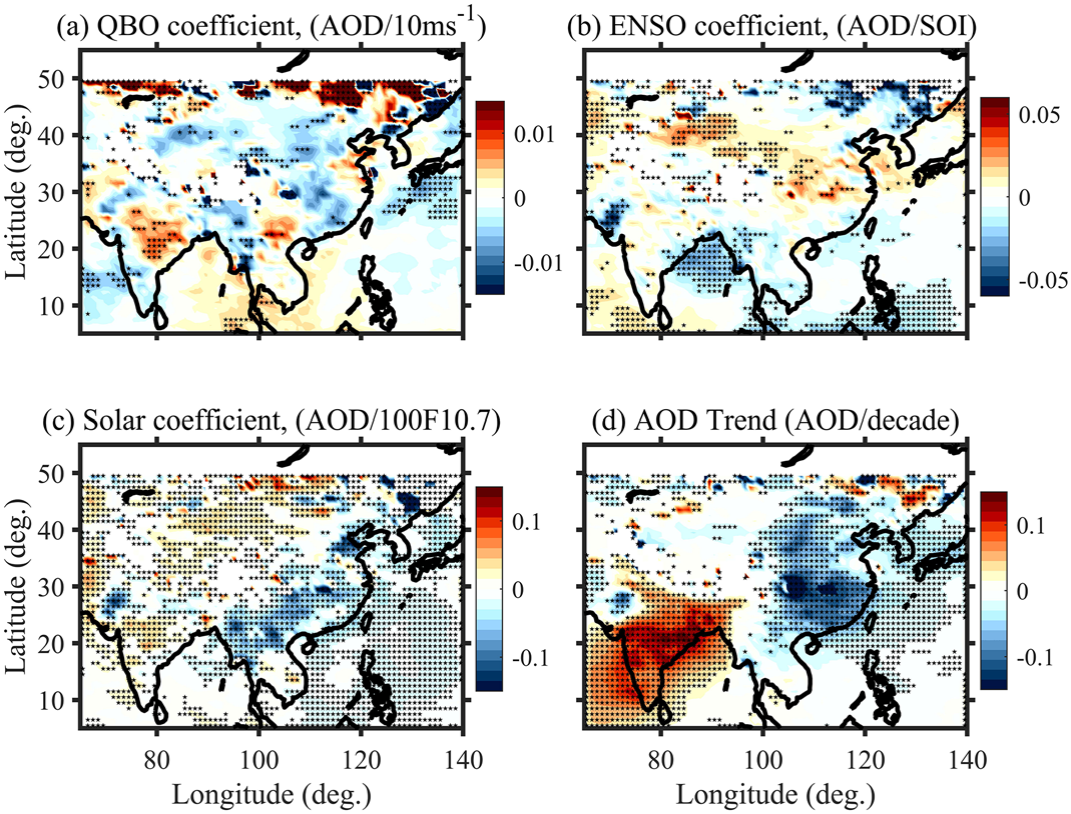
(**a**) QBO coefficient, (**b**) ENSO coefficient, (**c**) solar cycle coefficient obtained from the regression analysis applied on AOD measured by MODIS during 2001–2018. (**d**) Trends in AOD after removing the long-period oscillation contribution. Star marks show the trend that is statistically significant at 95% confidence level. The maps were created by using MATLAB software R2019b with mapping tool box (see https://in.mathworks.com/products/new_products/release2019b.html and https://www.mathworks.com/products/mapping.html).

In general, the time series of any atmospheric parameter contains natural oscillations like seasonal, semi-annual oscillation (SAO), annual oscillation (AO), Quasi-biennial oscillation (QBO), El Niño southern oscillation (ENSO), and solar cycle (SC) and these oscillations affect differently at different altitudes. Since AOD is the integral sum of aerosol extinction from the surface to the top of the atmosphere, it should also have profound effects. The effect of these signals has to be removed while calculating the long-term trends. In order to see the long-term trends in the aerosol concentrations over South and East Asia, monthly mean AOD at 1° × 1° obtained using MODIS during 2001–2018 is subjected to regression analysis mentioned in the methods section. Figure 2 shows the AOD response to QBO, ENSO and solar cycle. The regions with statistically significant trends at 95% confidence level are shown with star marks. Figure 2a shows AOD response to 30 hPa QBO wind which has a positive peak (0.01 QBO^−1^) over northeast India and southeast China and a similar magnitude of negative peak at rest of the places. AOD response to the ENSO is positive throughout South and East Asia except over northwestern and north BoB (Fig. 2b). AOD response to the solar cycle is mostly positive (negative) except over northwest (southeast) over India (China) (Fig. 2c). Thus, QBO, ENSO and solar cycle strongly influence the AODs over South and East Asia. Though the contribution of QBO and ENSO is expected in AOD but there may be a concern in the relation between AOD and solar cycle as there is no reported direct link between the two^25^. Further, 18 years long data may not be sufficient to elucidate the solar cycle effects as it requires at least two solar cycles (~ 22 years) of the data to draw a meaningful conclusion. To override this aspect, we also show linear trends observed in AOD in Supplementary Fig. S1. Trends in AOD show similar variations that are estimated using regression analysis revealing the robustness in the trends.

Trends in AOD after removing the natural oscillations during 2001–2018 are shown in Fig. 2d. The regions with statistically significant trends at 95% confidence level are shown with star marks. AOD shows a significant increasing trend (0.1 AOD/decade) over India including the Arabian sea and BoB except over the northwest region where a statistically significant decreasing trend is noticed. In contrast, a statistically significant decreasing trend (− 0.1 AOD/decade) in AOD is noticed particularly over east China. The trend is insignificant in the western and central parts of China. These trends are seen differently in different seasons (Supplementary Fig. S1). An increasing trend in AOD is noticed over IGP and the Indian mainland during the NH winter season (Supplementary Fig. S1b). In the spring season, coastal parts over BoB and southern India show an increasing trend in AOD (Supplementary Fig. S1c). Even during the Asian summer monsoon (NH summer), central India and eastern regions show a significant increasing trend in the AOD (Supplementary Fig. S1d). Similar to NH winter, the fall season (Supplementary Fig. S1e) also shows an increasing trend all over India. The decreasing trend in AOD is strongest in NH summer followed by spring and minimum in NH winter and the fall season over northwest India. Over China, the decreasing trend in AOD is strongest in NH summer and spring and restricts to east China during fall and trends in AODs are not significant during NH winter.

### Plausible reasons for increasing/decreasing trends in AOD

From the previous section, it is clear that a statistically significant increasing trend in AOD all over India except for north western parts (where a significant decreasing trend exists) and significant decreasing trends over east China is observed. In general, trends in aerosol concentrations depend on their emissions, Land Use Land Cover (LULC) and the background meteorological conditions^21^. In order to investigate the role of these on the trends in AODs, we make use of the Normalised Difference Vegetation Index (NDVI), surface temperature, convection/vertical velocity and rainfall. Since it is not possible to obtain trends in a similar manner that was presented for AOD for all the parameters mentioned above, we made a 5-year ensemble mean to represent past (2001–2005) and recent (2014–2018) periods and obtained their difference to discuss their trends. It is to be noted that the 5-year ensemble mean should take care of most of the long-period oscillations.

**Figure 3:**
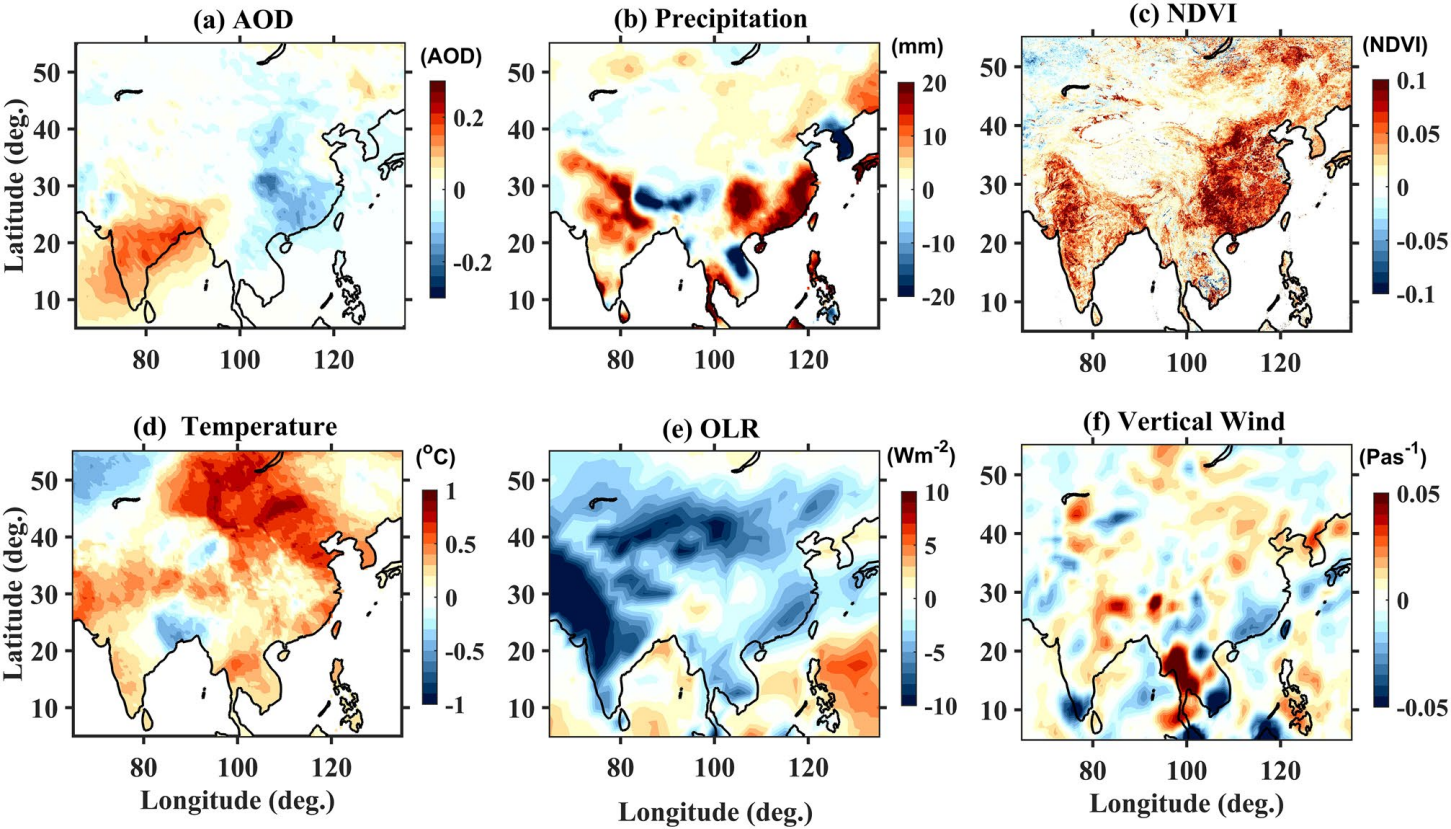
The 5-year ensemble mean difference (2014–2018 minus 2001–2005) observed in (**a**) AOD, (**b**) Precipitation, (**c**) NDVI, (**d**) Surface temperature, (**e**) OLR and (**f**) Pressure vertical velocity at 500 hPa. AOD and NDVI are obtained from MODIS, precipitation and surface temperature from CRU, OLR is taken from NOAA and pressure vertical velocity from ERA-Interim reanalysis data. The maps were created by using MATLAB software R2019b with mapping tool box (see https://in.mathworks.com/products/new_products/release2019b.html and https://www.mathworks.com/products/mapping.html).

Figure 3a shows the five-year ensemble mean difference in AOD between the recent (2014–2018) and the past (2001–2005) periods. The difference between the recent and the past periods in AOD matches well with the trends obtained through regression analysis (Fig. 2d), thus gives confidence in the ensemble mean difference analysis. The 5-year ensemble mean difference between the recent and the past in the precipitation [Climate Research Unit (CRU) data], NDVI (MODIS data) and surface temperature (CRU data) are shown in Fig. 3b–d, respectively. Their actual 5-year ensemble mean values are shown in Supplementary Figs. S2–S5. It is interesting to see an increasing pattern in NDVI over northwest parts of India (The Thar Desert and adjoining arid regions) and east China where significant decreasing trends in AODs are noticed. This increase in the vegetation can partly link to the increase in the rainfall (Fig. 3b). Thus, changes in AOD trends over the north-west region of India are linked to rainfall and vegetation. A similar increasing trend in NDVI and precipitation is also noticed in east China.

### Trends in the vertical distribution of aerosol concentrations

From the previous section, the dipole pattern in AODs is clearly seen with increasing trends over India and decreasing trends over China. How these trends vary in the vertical distribution of aerosol concentrations is not clear from these observations. In order to see the trends in the vertical distribution of aerosol concentrations, we make use of CALIOP observations. For this, we have estimated AOD within the boundary layer and free troposphere using the aerosol extinction profile obtained from CALIOP during 2006–2018. We have used an average height of 1.5 km (1 km) during the day (night) over India and 1 km over China as a boundary layer height, respectively. Reasons for opting these heights are mentioned in the methods section. In addition, simultaneous boundary layer altitude obtained from ERA-Interim reanalysis for both day and night overpass of CALIOP in each grid is also obtained. AOD is estimated from surface to the boundary layer altitude and boundary layer to the top of the atmosphere (free troposphere) for both day and night overpasses.

**Figure 4:**
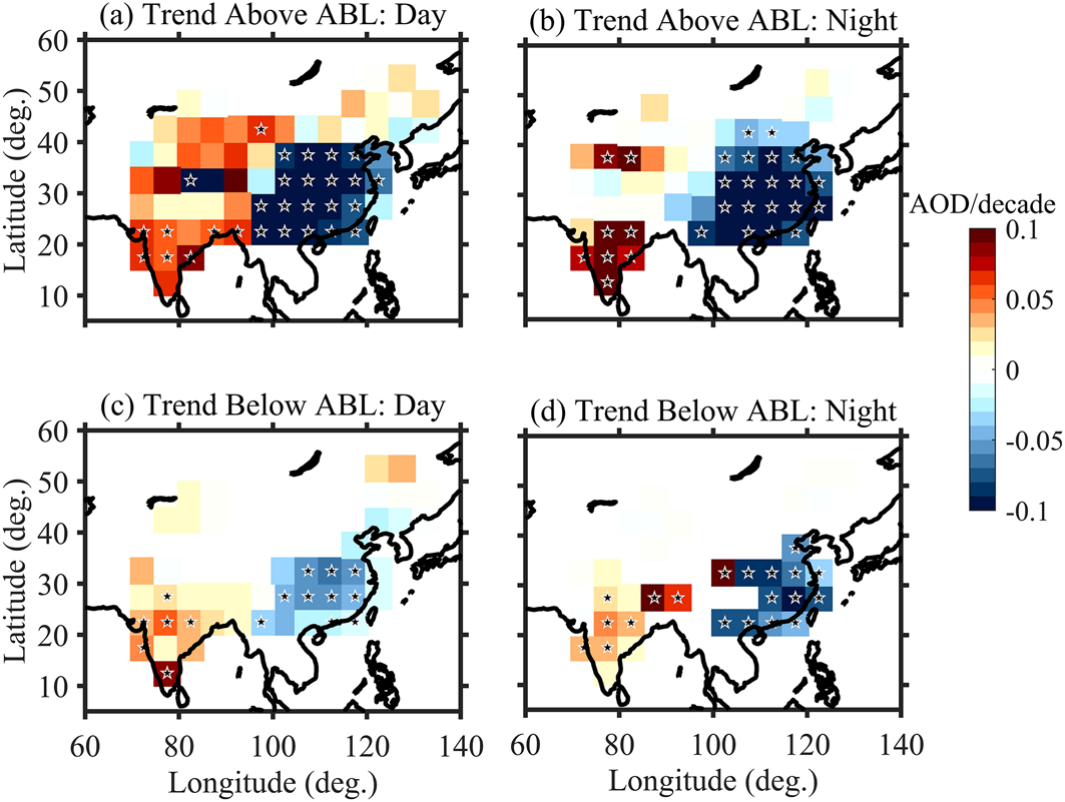
Trends in AOD observed in the free troposphere during (**a**) day time and (**b**) night time over south and East Asia obtained using CALIOP measurements during 2006–2018. (**c**) and (**d**) same as (**a**) and (**b**) but for within the boundary layer. Star marks show the trend that is statistically significant at 95% confidence level. The maps were created by using MATLAB software R2019b with mapping tool box (see https://in.mathworks.com/products/new_products/release2019b.html and https://www.mathworks.com/products/mapping.html).

Trends in AOD within the boundary layer and free troposphere for both day and night overpass of CALIOP after removing the contributions of seasonal, QBO, ENSO and solar cycle are shown in Fig. 4. Statistically significant trends at 95% confidence level are marked with a star. A statistically significant increasing trend in AOD within the boundary layer is seen over India at only a few places (Central/south India) during day time (Fig. 4c) and night time (Fig. 4d). The majority of regions do not show any significant trends. However, a statistically significant decreasing trend in AOD is seen over east China. Similar features are also reported over China by Dong et al*.*^26^. On the other hand, in the free troposphere, significant increasing trends are seen during both day (Fig. 4a) and night times (Fig. 4b) over most parts of India. Interestingly, a significant decreasing trend in the AOD is seen even in the free troposphere over China irrespective of day and night. Thus, it is clear that trends in aerosol vertical distribution are also changing in changing climate.

Transport (both vertical and horizontal) induced by the dynamics should be one of the plausible reasons for the observed changes in the vertical distribution of aerosols. Several reports mentioned about the increase in convection in the tropical regions in changing climate which is clear from Fig. 3e. In recent years, the frequency of occurrence of deep convective clouds has increased over tropical land^27^. Using long-term Atmospheric Infrared Sounder measurements, an increasing trend in the tropical deep convection over land is being reported^28^. Further, intense meso-scale convective systems in which cloud-top temperatures lower than − 70 °C (~ 150 hPa) have been largely increased over the Sahel since 1982^29^. This advocates that tropical deep convection penetrating into the Tropical Tropopause Layer (TTL) mostly increased, which is consistent with the present study. This increase in convection mainly due to global warming (increase in surface temperature shown in Fig. 3d) should also echo in the vertical velocity as they are inter-linked. The 5-year ensemble mean difference between the recent and past in Outgoing Long-wave Radiation (OLR) and pressure vertical velocity at 500 hPa is shown in Fig. 3e,f, respectively. Their original 5-year ensemble mean values are shown in Supplementary Figs. S6 and S7. Positive (negative) values in pressure vertical velocity represent downward (upward) wind. Note that OLR can be taken as a proxy for tropical convection (low values of OLR suggest high cloud top height and thus deep convection) and pressure vertical velocity at 500 hPa represents the vertical velocity at mid-troposphere. The choice of 500 hPa is selected based on a study by Park and Allen, where it was shown that this level represents well above the boundary layer^30^. An increase in the convection throughout India with the strongest over the central and northwest parts of India and northeast China is noticed (Fig. 3e). An increase in convection can lift more boundary layer pollutants to the free troposphere and this might be one of the reasons for the observed increase in free troposphere aerosol concentrations. The increase in the upward vertical wind (Fig. 3f) also supports this argument. Thus, convective mass flux is one of the main mechanisms for lifting boundary layer pollutants to the free troposphere^30^. Since vast region is considered and the background conditions may not the same throughout, we further made spatial correlation analysis between the AOD and temperature, precipitation, vertical wind, convection (OLR) and NDVI and is shown in Supplementary Fig. S8. In general, AOD shows a positive correlation with temperature (Supplementary Fig. S8d) and precipitation (Supplementary Fig. S8b) with strongest (0.8–0.9) over northwest India, central and northeast China. AOD shows a negative correlation (0.6–0.7) with vertical wind (Supplementary Fig. S8f) over most of the regions of India except over IGP. A similar relation is seen in OLR (Supplementary Fig. S8e) over India but it shows a positive correlation over northeast China. NDVI is negatively correlated with AOD over northwest and IGP regions and southeast China (Supplementary Fig. S8c).

We also checked trends in the boundary layer altitude and noticed an increase of about 180 m in the last 18 years over central India and this change may also contribute to the AOD estimation within and above the boundary layer altitude. In order to check this, we obtained AOD while considering boundary layer altitude from ERA-Interim during both day and night and are shown in Supplementary Fig. S9. One can notice similar trends (except some changes during night times where a smaller number of statistically significant points exist) revealing that changes in the boundary layer itself are not significant.

## Summary and discussion

Changing patterns in aerosol vertical distribution over South and East Asia is presented using long-term satellite (active and passive) remote sensing measurements. Consistent with earlier reports (Samset et al*.*), dipole pattern in AODs i.e., statistically significant increasing trends (0.1 AOD/decade) over India (in particular central India and Arabian Sea) and decreasing trends over China are observed in long-term MODIS observations^5^. Several earlier studies reported higher aerosol loading over the IGP region than any other parts of India but the present study reveals a significant increase in the aerosol loading over central India in recent periods (2014–2018). Though similar features are also reported during the NH winter season over India by Thomas et al.^11^, our study shows an increase in aerosol concentrations irrespective of the season, including the NH summer season where chances of wet scavenging (due to monsoon activity) are high.

Increasing/decreasing trends in aerosol concentrations over a given region depends on their sources and sinks. Aerosol sinks over a given place are dictated by both wet (rainfall) and dry removal (diffusion and transport) where the former is most effective during the Asian summer monsoon (NH summer) but the latter persists throughout the year over India. Though the overall increase in rainfall is being noticed (Fig. 3b) in the recent period, where chances of wet removal of aerosol are high, an increase in AOD is observed over India. This is mainly due to an increase in the dry phases being reported over India in a changing climate^31,32^. This will reduce the chances of wet removal (in-cloud and below cloud scavenging) finally leading to an increase in aerosol concentrations^33^. The warming trend observed in surface temperatures over most parts of India (Fig. 3d) generally associated with an increase in land aridity, which in turn may affect the burden of atmospheric aerosols. Aerosol sources include extensive crop residue burning and forest fires besides transport, rapid urbanization and industrialization^24^. An increase of 20% in fire counts over central India has been reported between past and recent periods^11^. Thus, higher aerosol concentrations over central India in the recent periods can be attributed to the increase in the fire emissions. Aerosol concentrations from these fires can extend over several parts of India through favourable wind conditions. Banerjee et al. showed that 50% of the total aerosol (dust) loading over BoB through the IGP region during the monsoon period is from regional sources (the Thar Desert and adjoining arid regions)^34^. Whereas dust transported from remote sources (southwest Asia and northeast Africa) dominates throughout the year. Therefore, the observed increase in the recent aerosol loading over central and northeast India can be a combined result of enhanced fire emissions and long-range transport of aerosols.

However, over the northwest region of India, significant decreasing trends in AODs are observed (Fig. 3a). These decreasing trends are attributed to increasing trends in rainfall (Fig. 3b) leading to an increase in NDVI (Fig. 3c) over this region. In recent years, desert areas in the northwest India are being converted to crop-land areas, as a result, an increasing trend in NDVI is being noticed^35,36^. It is understood here that enhanced vegetation leads to an increase in rainfall^37^. Thus, the decrease in aerosol concentrations over northwest India can be linked to the increase in wet removal (in-cloud and below-cloud scavenging) associated with the increase in rainfall and an increase in vegetation.

Statistically significant effects of long-period oscillations (QBO, ENSO and Solar Cycle) are also observed in AODs. Interestingly, a shift in the aerosol vertical distribution over India is found using long-term CALIOP observations where weak increasing trends in boundary layer aerosol at only a few regions and statistically significant increasing trends in the free tropospheric aerosols are noticed during both day and night times over India (Fig. 4). Using long-term surface observations, decreasing trends in the boundary layer (BC) aerosol over Gadanki is confirmed by Ravi Kiran et al*.*^12^ and over several parts of India by Manoj et al*.*^13^. The latter study also revealed a shift in the aerosol vertical distribution i.e., an increasing trend in the free tropospheric aerosol concentrations. Further, the existence of elevated aerosol layers during recent periods in satellite measurements are confirmed by ground-based remote sensing over India^22^.

Meteorology and dynamics play an important role in changing patterns of aerosol distributions. The increase in free tropospheric aerosol concentrations are attributed to the increase in tropical convection (Fig. 3e) in recent periods due to an increase in surface temperature (Fig. 3d) (as a part of global warming) leading to the stronger vertical velocities (Fig. 3f) in the free troposphere. Thus, the boundary layer pollutants can be easily lifted to the free troposphere through convection and upward vertical velocities leading to the enhancement in free tropospheric aerosol concentrations. A study by Park and Allen^30^ also support this contention where they observed higher convective mass flux associated with more precipitation and more aerosol vertical dispersivity including more aerosol concentrations above 500 hPa over tropics. However, irrespective of the altitude region, decreasing trends in aerosols are observed over China. Though stronger vertical velocities in the free troposphere are also observed over China, since there is a drastic decrease in boundary layer pollutants itself^14–16^, the effect of this is not seen on the free troposphere aerosol concentrations. Further, an increase in the precipitation (Fig. 3b) leading to the increase in NDVI in the recent period (Fig. 3c) might also played role in the clean-up of the aerosol over east China. Due to stringent ‘clean air actions’ taken by China in the last decade, 50–60% reduction in the aerosol concentrations is being reported^10^. No reductions in anthropogenic aerosol emissions over India are being reported^37^. Further, more and more dry phases are being reported over India in a changing climate^31,32^. In addition, due to the increasing frequency of droughts, South Asia underwent a widespread declining trend in its total seasonal monsoon precipitation (~ 7% from 1951 to the present). This is largely blamed on anthropogenic aerosols as they generally reduce temperatures over land, resulting in weaker land-sea thermal contrast^32^. This increase in dry phases also leads to an increase in aerosol concentration in the free troposphere due to the persistence of convection even during dry phases lift boundary layer aerosol to the free troposphere. In addition, long-range transport through low-level jet continues even during break phases that can transport pollutants from long distances (through the Arabian sea)^22,23^. Thus, the aerosol vertical distribution over the Indian region is significantly affected by the vertical transport through convection and precipitation.

Using novel observations and reanalysis model data sets, in the present study we demonstrate that meteorology and dynamics play a major role in changing patterns of aerosol vertical distribution over the South and East Asian region. Note that the increasing trend in free tropospheric aerosol over India has important consequences on the background meteorology and dynamics besides their radiative forcing at those altitudes or below. Depending on the aerosol types, they can modify the cloud condensation nuclei (CCN) activity directly and the background instability^38^ indirectly, thus affecting convection and/or precipitation processes. Chemical compositions at different altitudes need to be obtained and checked for any change in the trends. Further, model simulations are required to quantify the contribution of vertical and long-range transport to the total aerosol loading over these regions.

## Data and methods

### MODIS measurements

We make use of gridded (1° × 1°) monthly mean MODIS observations obtained during 2001–2018 for investigating trends in AOD over South and East Asia. Aerosol products derived from the MODIS satellite are proven to be good over oceans, dark and bright land surfaces due to significant modifications made in the aerosol retrieval algorithms. Over dark surfaces (vegetative land and oceans), AOD is retrieved using dark-target (DT) algorithm and for bright surfaces deep blue (DB) algorithms are used^39^. Complete details of DT and DB algorithms are provided in Levy et al*.*^40^. In this study, we have used MODIS-Terra merged DT and DB AOD at 550 nm for the land and ocean (MOD08_M3) C6.1 Level 3 monthly AOD. These C6.1 Level 3 observations from this instrument have a spatial resolution of 1 km (or less depending on the band) at nadir with a swath of 2330 km and have 36 spectral bands^41^. In MODIS retrieval algorithm, AOD is first determined at a nominal 1 km × 1 km spatial resolution for clear sky and snow free pixels. Later it is averaged to 10 km × 10 km pixel scale by using quality assurance (QA) flag with QA = 3 indicating the retrievals are at the highest confidence and provides Level 3 products at (1° × 1°) daily, 8-day and monthly temporal resolutions. Version C6.1 data requires valid retrievals at least three days in a month for providing monthly products. Sayer et al*.* have discussed the uncertainty in the AOD by validating MODIS AOD at 550 nm data with AErosol RObotic NETwork (AERONET) from 60 sites^39^. Studies also reported that combined DT and DB (DTB) products are accurately capturing the exact aerosol changes and recommended for aerosol studies at a global scale^41^. This data is available from https://modis.gsfc.nasa.gov.

We also make use of Normalised Difference Vegetation Index (NDVI) from MODIS VI (MOD13) to investigate the trends in vegetation. NDVI derived from MODIS has a consistent relationship with vegetation biomass and communities^42^. These products are available at multiple spatial resolutions for every 16 days at a given location. Level 3 daily data is retrieved from bidirectional surface reflectance after atmospheric corrections. For the present work, we have used Level 3 Monthly NDVI Global (MOD13C2) data with a spatial resolution of 0.05° covering both India and China obtained from 2001 to 2018. This data is available from https://modis.gsfc.nasa.gov.

### CALIOP on-board CALIPSO measurements

For investigating the vertical distribution of aerosols, we made use of CALIOP on-board CALIPSO measurements obtained during 2006–2018. We used version 4.10 level 2 aerosol layer products. CALIOP is a two-wavelength (1064 nm and 532 nm), nadir viewing sun synchronous polarization Lidar with a pulse energy of 110 mJ and a pulse repetition rate of 20.25 Hz^19^. CALIOP provides the vertical profile of atmospheric particles at 532 and 1064 nm near nadir during both day and night. CALIOP path profile is available with 5 km horizontal, 60 m vertical and 16-days temporal resolution. This data is available from https://eosweb.larc.nasa.gov/project/calipso/calipso_table. Complete details of the CALIOP instrument, data products and data acquisition can be found in Anselmo^43^ and retrieval algorithms of aerosol extinction and backscatter coefficients are given in Young and Vaughan^44^. We used the EXT_QC flag to remove the false retrieval from TBC values in the level 2 aerosol layers^19^. In general, aerosol extinction is very sensitive to lidar ratio and multiple scattering factors^45^. Version 3 products had lot of ambiguity in choosing the lidar ratio and it is resolved in Version 4 through extensive upgrades in the retrieval algorithms^45^. Hence, this data will produce the best results that are more representative of the actual condition of the atmosphere. Still, this algorithm has compromised by the low Signal to Noise Ratio (SNR) during day time measurements that may lead to weaker backscatter signals. Uncertainties in the aerosol extinction retrieval are discussed in Young et al*.*^44^. CALIOP products are validated with independent ground-based lidar measurements^46,47^. Since the near surface AOD estimation from CALIOP has large uncertainty due to strong attenuation of backscattering signals in the boundary layer, we have discarded measurements of the first few range bins (< 200 m) while estimating the AOD within the boundary layer.

Comparison between AOD obtained from satellite-borne (MODIS and CALIOP) and ground-based remote sensing instruments (Lidar) revealed very good consistency^23^. Several studies already reported good consistency between the MODIS AOD and AOD obtained from AERONET measurements^48,49^. We further compared MODIS AOD with the AERONET observations over Kanpur (26.5° N, 80.2° E) where continuous observations are available during 2000–2018 and is shown in Supplementary Fig. S10. A very good correlation of 0.75 is noticed. Thus, overall features obtained from these two satellite measurements compared well with that obtained using ground-based lidar observations^23^ and also independent verification using AERONET observations (Supplementary Fig. S10).

### Climate research unit (CRU) data

The monthly gridded surface temperature data from CRU version TS4.0 is used in the present study for the years 2001–2018 covering South and East Asia^50^. This data is available with 0.5° × 0.5° latitude/longitude grid over land regions across the globe. This data is produced by CRU at the University of East Anglia using more than 4000 weather stations data globally. Consistency in this data over India is checked using the India Meteorological Department (IMD) network and found that both reproduce well all the major features^51^.

### ERA-Interim Reanalysis data products

The ERA-Interim global atmospheric reanalysis products were provided by the European Centre for Medium-Range Weather Forecasts (ECMWF). For the present study, we make use of boundary layer altitude and vertical wind over the Asian region from these reanalysis^52^. These gridded products are available at every 6 h (http://dataportal.ecmwf.int/data/d/interim_daily/levtype=pl/) at different spatial resolutions. Complete documentation regarding the data is available from the website http://www.ecmwf.int/publications/newsletters.

### NOAA-interpolated outgoing long wave radiation (OLR)

We also make use of the National Oceanic and Atmospheric Administration (NOAA) interpolated Outgoing Long wave radiation (OLR) 2.5° × 2.5° daily mean data product during 2001–2018. The data and description of the data are available from the website https://www.esrl.noaa.gov/psd/data/gridded/data.interp_OLR.html#detail. Monthly mean OLR less than 240 W/m^2^ is only considered as convection.

## Methodology

### Estimation of long-term trends

For estimating the long-term trends, multivariate regression analysis is applied to the time series of monthly mean AOD in each grid over South and East Asia. The general expression of the regression model is given by Randel and Cobb^53^,

1 $${\text{T}}(t,z) = \, \alpha (z) + \, \beta \left( z \right)t + \, \gamma (z){\text{QBO}}(t) + \, \delta (z){\text{Solar}}(t) + \, \varepsilon (z){\text{ENSO}}(t) + {\text{ resid}}\left( {\text{t}} \right).$$

The coefficients α, β, γ, δ and ε are calculated using the following harmonic expression,

2 $$\alpha \left( z \right) = A_{o} + \mathop \sum \limits_{i = 1}^{3} \left[ {A_{i} \times cos\omega_{i} t + B_{i} \times sin\omega_{i} t} \right],$$

where ω_i_ = 2πi/12.

If N is the length of these data, S is the sum of the square of residuals, M is the total number of regression constants and X is the input data matrix, the error in the coefficients is given by,

3 $$\sigma = \sqrt {\frac{S}{N - M} (X^{T} X)^{ - 1} } .$$

The 30 hPa monthly mean QBO zonal wind (m s^−1^) at Singapore (1° N, 104° E) is used as QBO proxy QBO(t)^54^. This data is obtained from http://www.geo.fu-berlin.de/met/ag/strat/produkte/qbo. Further, the monthly mean radio emissions from the sun at F10.7 cm wavelength from Ottawa is used as a proxy for the solar cycle, solar(t)^54^. This data is obtained from ftp://ftp.geolab.nrcan.gc.ca/data/solar_flux/monthly_averages/maver.txt. Southern oscillation index (SOI) is used as El Niño-Southern oscillation proxy, ENSO(t)^54^. This data is obtained from http://www.cpc.ncep.noaa.gov/data/indices/soi.

### Uncertainty in the boundary layer height

Estimation of accurate Atmospheric Boundary Layer (ABL) height is essential to investigate the trends within ABL and free troposphere. ABL height can be estimated from the backscattered profiles of CALIOP but also suffers from serious limitations. In the study by Zhang et al*.* it was mentioned that ABL height estimated by CALIOP underestimates heavily and found a correlation of 0.59 and 0.65 only when compared with ABL height obtained from radiosondes over China^55^. In addition, it is also not possible to obtain ABL height from backscatter profiles at times and zones when the aerosol concentrations are low. In fact, a strong gradient in backscatter occurs when there is a temperature inversion. At this inversion, most of the pollutants/water vapour will be trapped and hence strong gradient will be observed. Thus, ABL height estimated by temperature or water vapour or combined parameters (refractivity or virtual potential temperature) is found more useful^56,57^. However, as mentioned in these papers, a difference of 500–900 m exists when compared with ABL altitude from network of radiosonde over both India and China. Nevertheless, monthly mean ABL height obtained from the radiosonde network over India (see Supplementary Fig. S11) and China (Guo et al*.*) very recently reveals that it varies from 1.3–1.5 km (over India) and from 0.9–1.1 km (over China) on an average during 2000–2015^58^. Thus, an average altitude of 1.5 km (1 km) during the day (night) over India and 1 km over China is considered as ABL height and estimated AOD below and above in Fig. 4. We also considered day and night variation in the ABL height from ERA-Interim and AOD estimated while considering the varying ABL height and is shown in Supplementary Fig. S9. Though some differences are noticed particularly during night times, but the overall features remain same. Further, except small underestimation/overestimation in different seasons, ABL height estimated using radiosonde and ERA-Interim matches well over China^59^. Thus, uncertainty in the ABL height will not impact the observed features in a significant manner.

## Supplementary Information

Supplementary Information.

## Data Availability

Satellite (MODIS and CALIOP), NOAA OLR, CRU and model reanalysis (ERA-Interim) datasets are freely accessible to the public from their respective websites.
