# Supplementary material for: Changing patterns in aerosol vertical distribution over South and East Asia
Source: Sci Rep. 2021 Jan 11;11:308. doi: 10.1038/s41598-020-79361-4 (PMC7801640; doi:10.1038/s41598-020-79361-4)
Supplement: Supplementary file 1 — Supplementary Information. [file 41598_2020_79361_MOESM1_ESM.docx]

**Changing patterns in Aerosol Vertical Distribution Over South and East Asia**

M. Venkat Ratnam^1*^, P. Prasad^1^, S.T. Akhil Raj^1^, M. Roja Raman^2^ and Ghouse Basha^1^

^1^National Atmospheric Research Laboratory, Gadanki-517112, India

^2^Research Centre for Environmental Changes, Academia Sinica, Taipei 11529, Taiwan

*Email: vratnam@narl.gov.in

**Supplementary Figures**


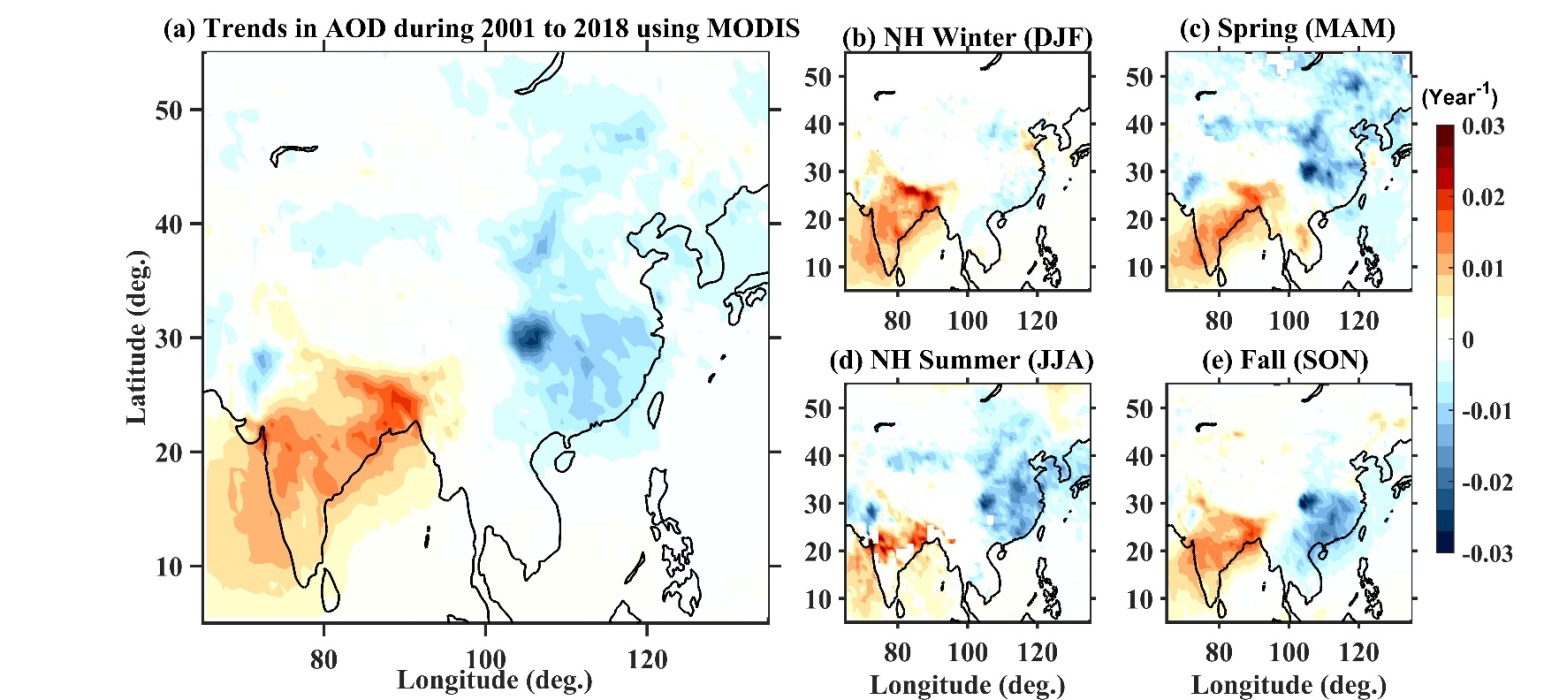


**Figure S1.** Trends in AOD over South and East Asia observed using MODIS during 2001-2018. Seasonal trends in AOD observed during (b) NH winter (DJF), (c) spring (MAM), (d) NH summer (JJA) and (e) Fall (SON) seasons during the same period. The maps were created by using MATLAB software R2019b with mapping tool box (see <https://in.mathworks.com/products/new_products/release2019b.html> and <https://www.mathworks.com/products/mapping.html>).


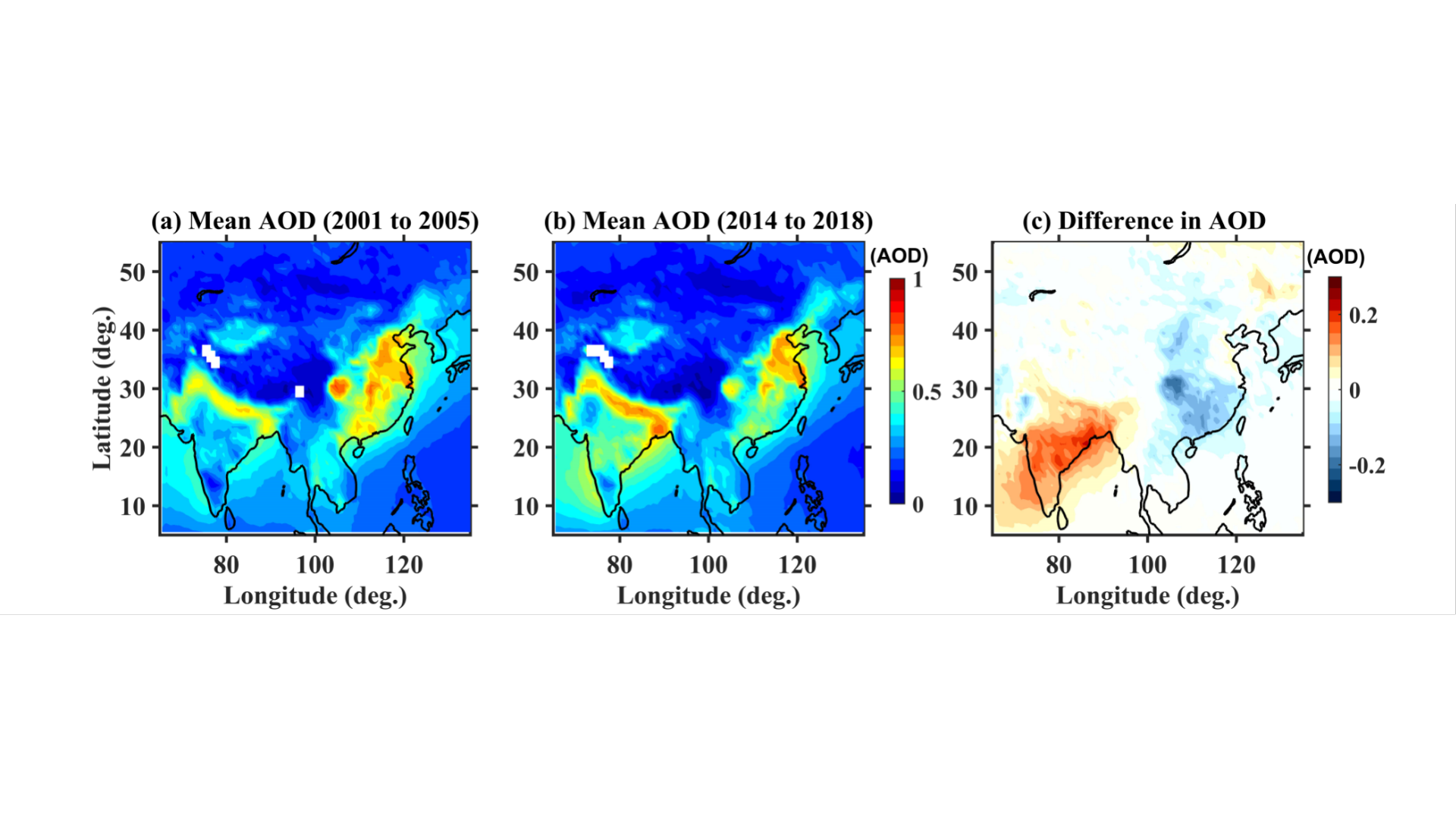


**Figure S2:** Five-year ensembles of AOD observed by MODIS during (a) 2001-2005 (past years) and (b) 2014-2018 (recent years) and the (c) difference between the recent and past years. The maps were created by using MATLAB software R2019b with mapping tool box (see <https://in.mathworks.com/products/new_products/release2019b.html> and <https://www.mathworks.com/products/mapping.html>).


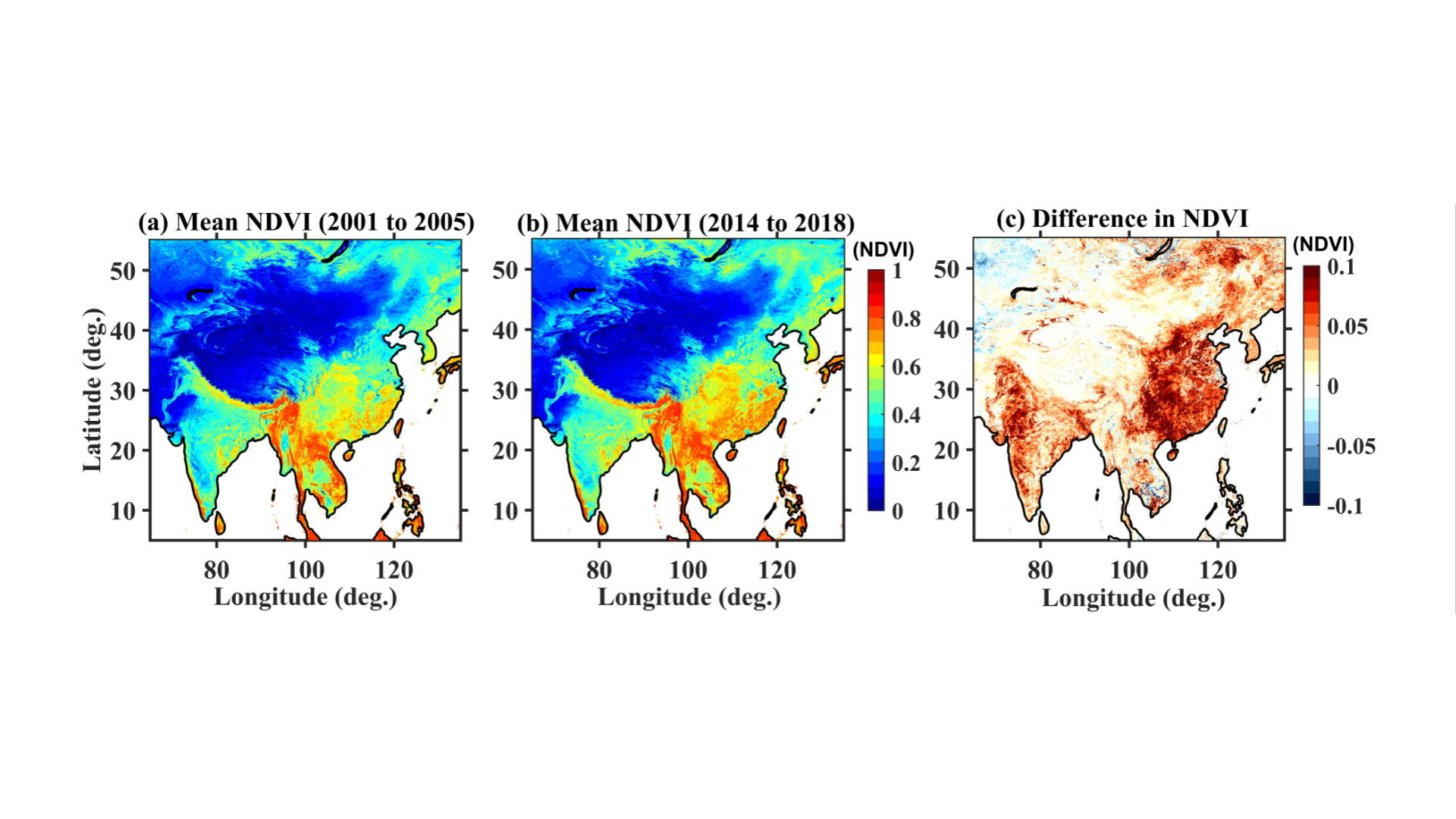


**Figure S3:** Same as Figure S2 but for NDVI. The maps were created by using MATLAB software R2019b with mapping tool box (see <https://in.mathworks.com/products/new_products/release2019b.html> and <https://www.mathworks.com/products/mapping.html>).


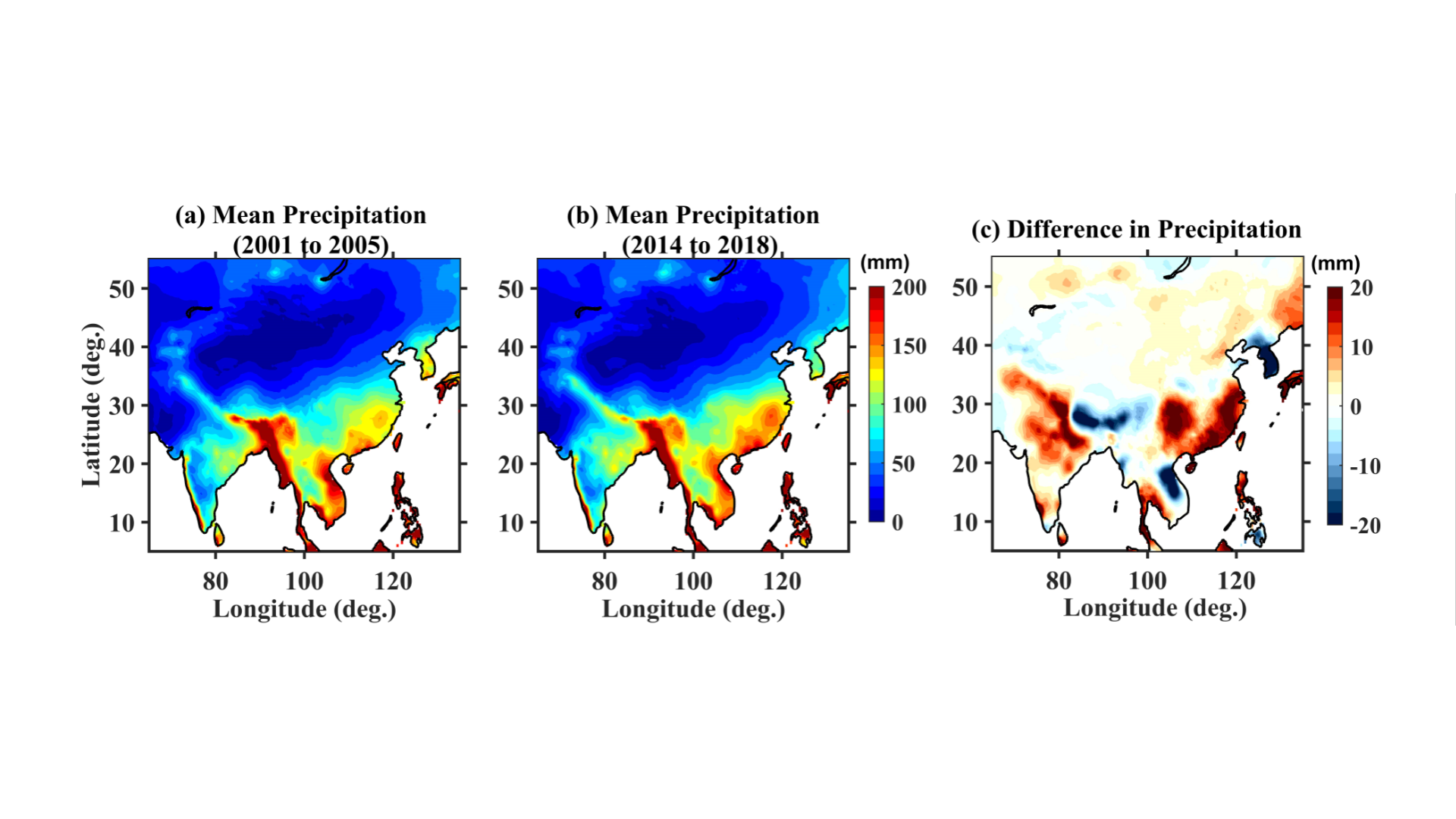


**Figure S4:** Same as Figure S2 but for precipitation obtained from CRU network. The maps were created by using MATLAB software R2019b with mapping tool box (see <https://in.mathworks.com/products/new_products/release2019b.html> and <https://www.mathworks.com/products/mapping.html>).


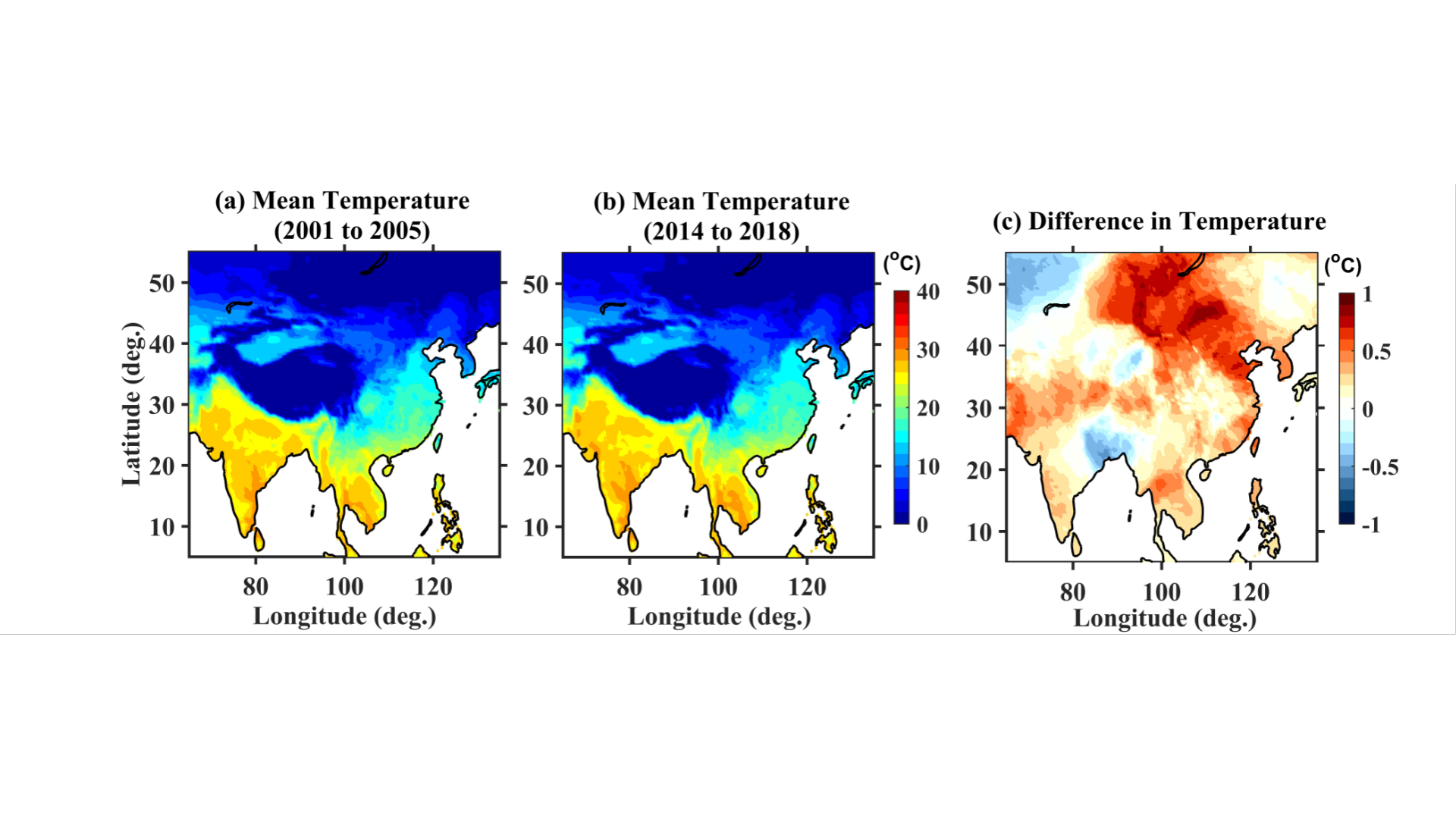


**Figure S5:** Same as Figure S2 but for temperature obtained from CRU network. The maps were created by using MATLAB software R2019b with mapping tool box (see <https://in.mathworks.com/products/new_products/release2019b.html> and <https://www.mathworks.com/products/mapping.html>).


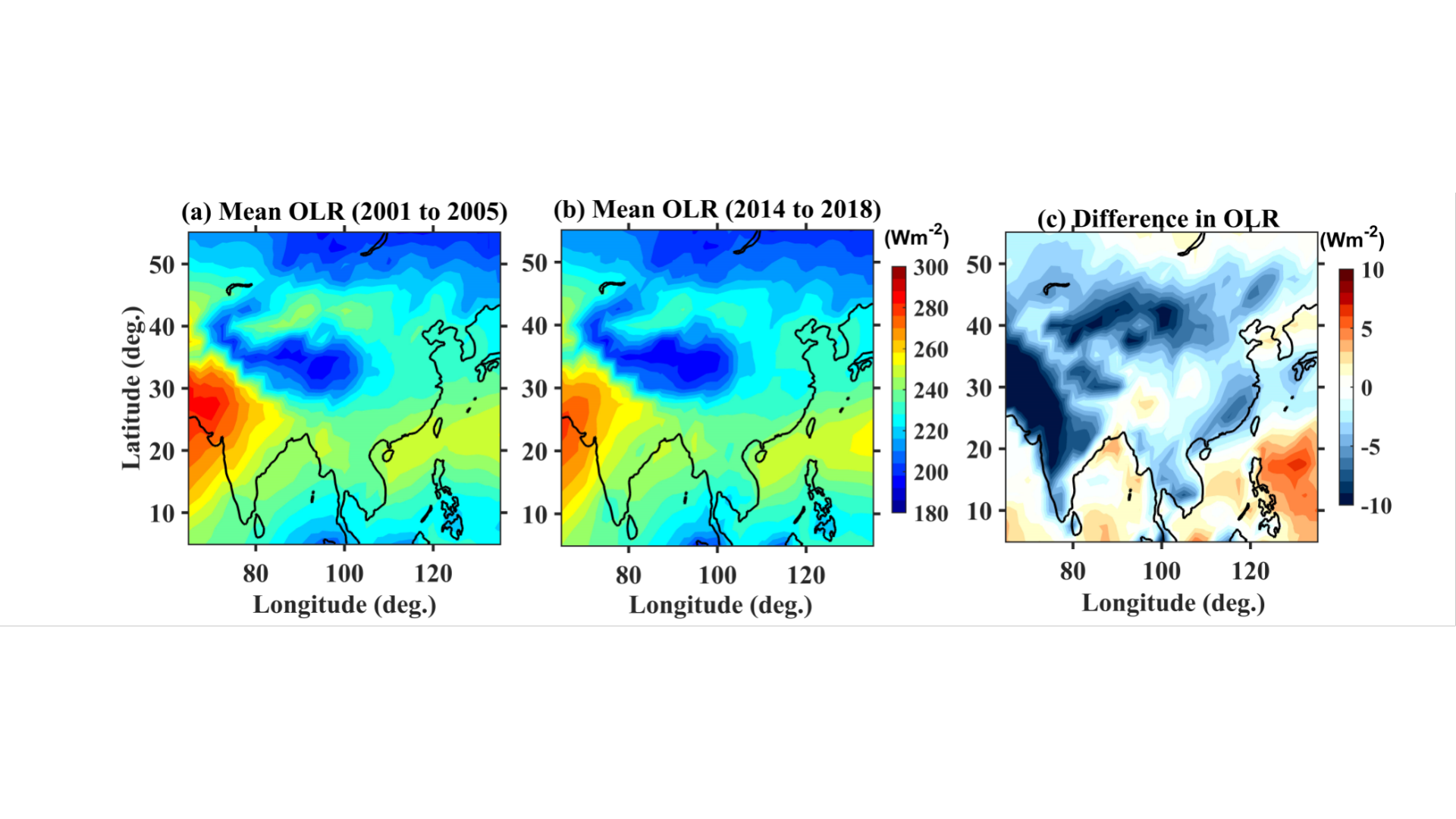


**Figure S6:** Same as Figure S2 but for OLR obtained from NOAA data. The maps were created by using MATLAB software R2019b with mapping tool box (see <https://in.mathworks.com/products/new_products/release2019b.html> and <https://www.mathworks.com/products/mapping.html>).


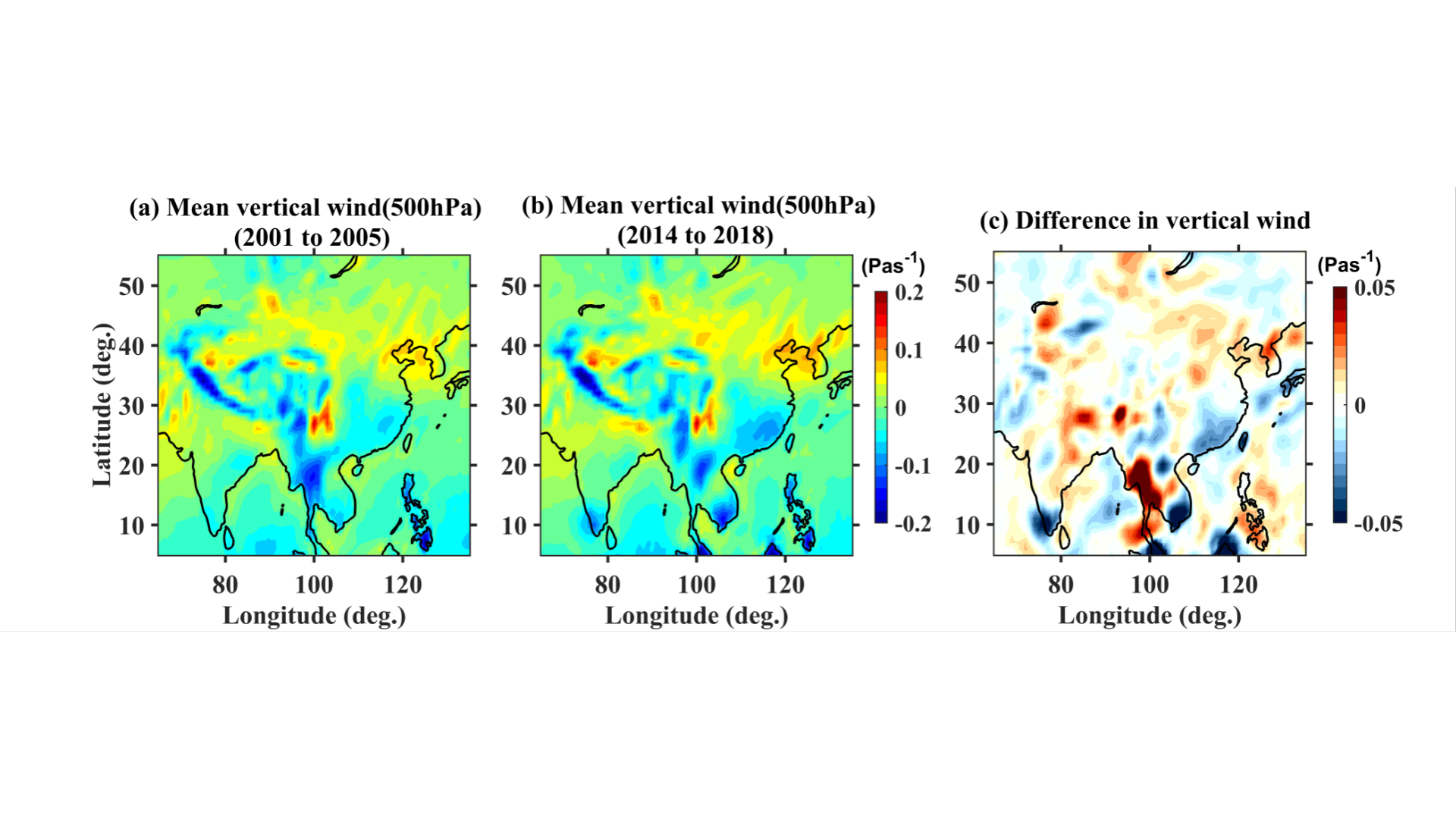


**Figure S7:** Same as Figure S2 but for pressure vertical velocity at 500 hPa obtained from ERA-Interim reanalysis data. The maps were created by using MATLAB software R2019b with mapping tool box (see <https://in.mathworks.com/products/new_products/release2019b.html> and <https://www.mathworks.com/products/mapping.html>).


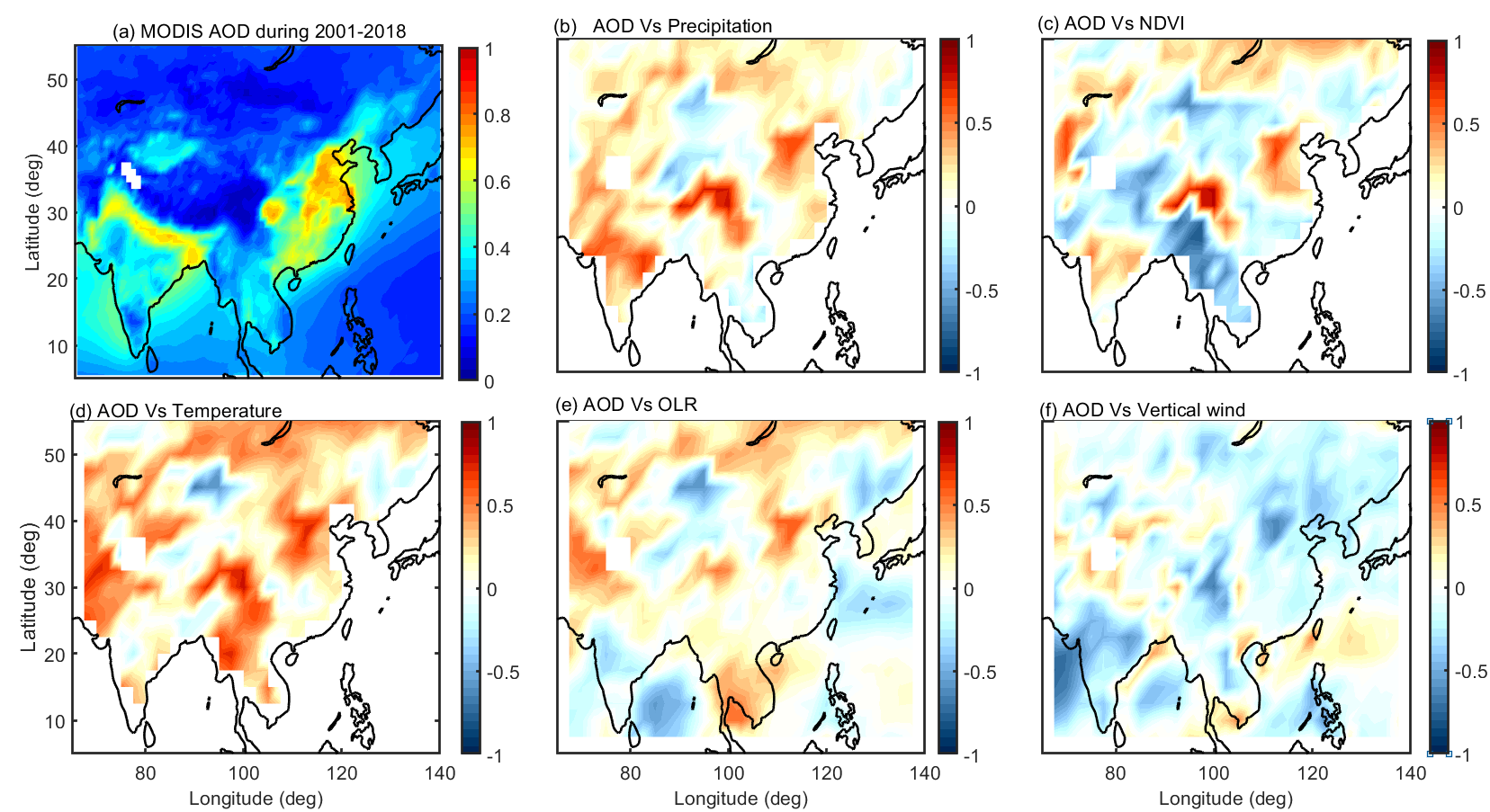


**Figure S8:** (a) Aerosol Optical Depth (AOD) climatology over South and East Asia observed using MODIS averaged during 2001-2018. Spatial correlation between AOD and (b) precipitation, (c) NDVI, (d) temperature, (e) OLR and (f) vertical wind obtained during 2001-2018. The maps were created by using MATLAB software R2019b with mapping tool box (see <https://in.mathworks.com/products/new_products/release2019b.html> and <https://www.mathworks.com/products/mapping.html>).


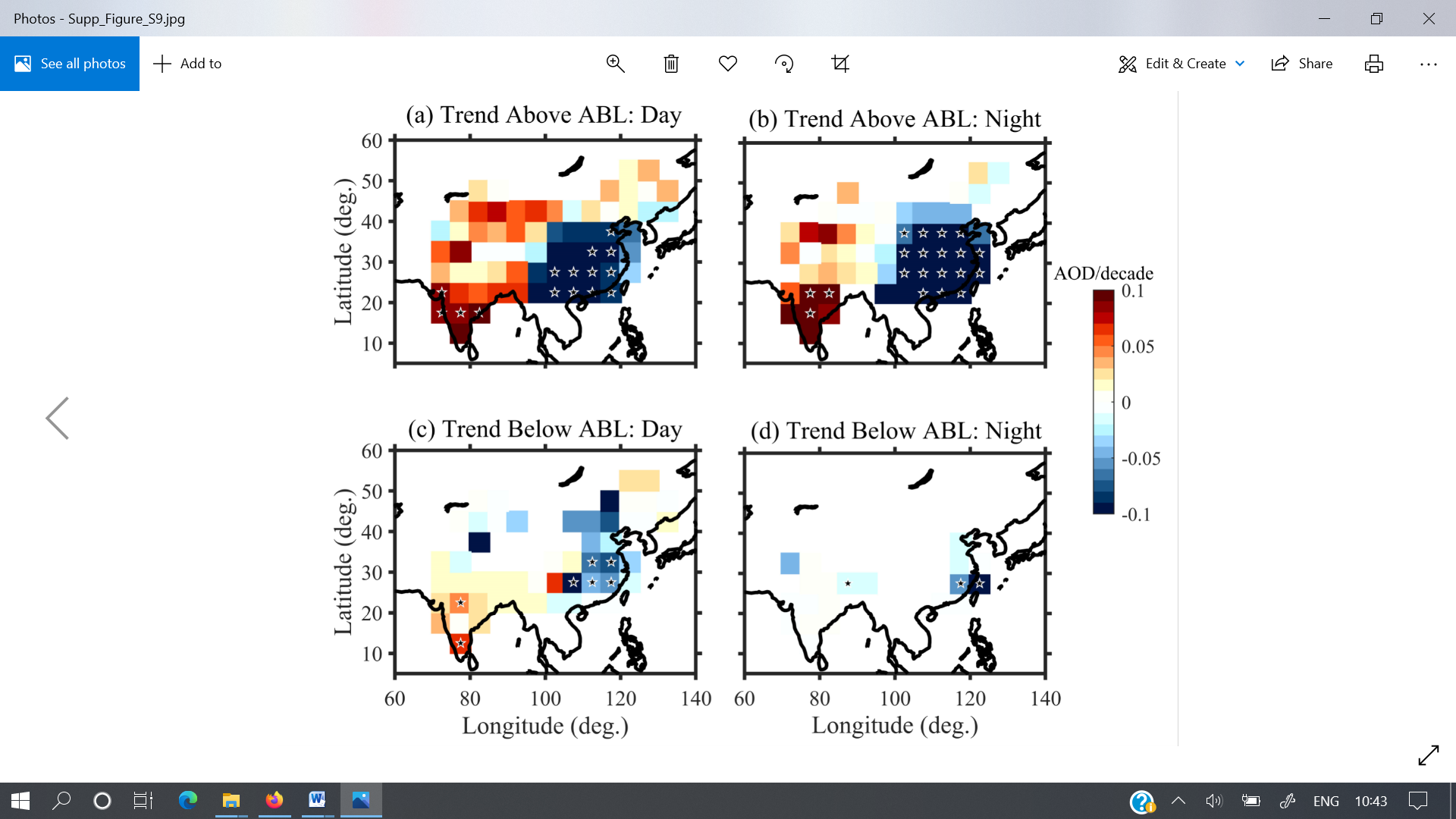


**Figure S9.** Same as Figure 4 but using the boundary layer altitude obtained from ERA-Interim reanalysis data. Star symbol denotes trend is statistically significant at 95% confidence level. The maps were created by using MATLAB software R2019b with mapping tool box (see <https://in.mathworks.com/products/new_products/release2019b.html> and <https://www.mathworks.com/products/mapping.html>).


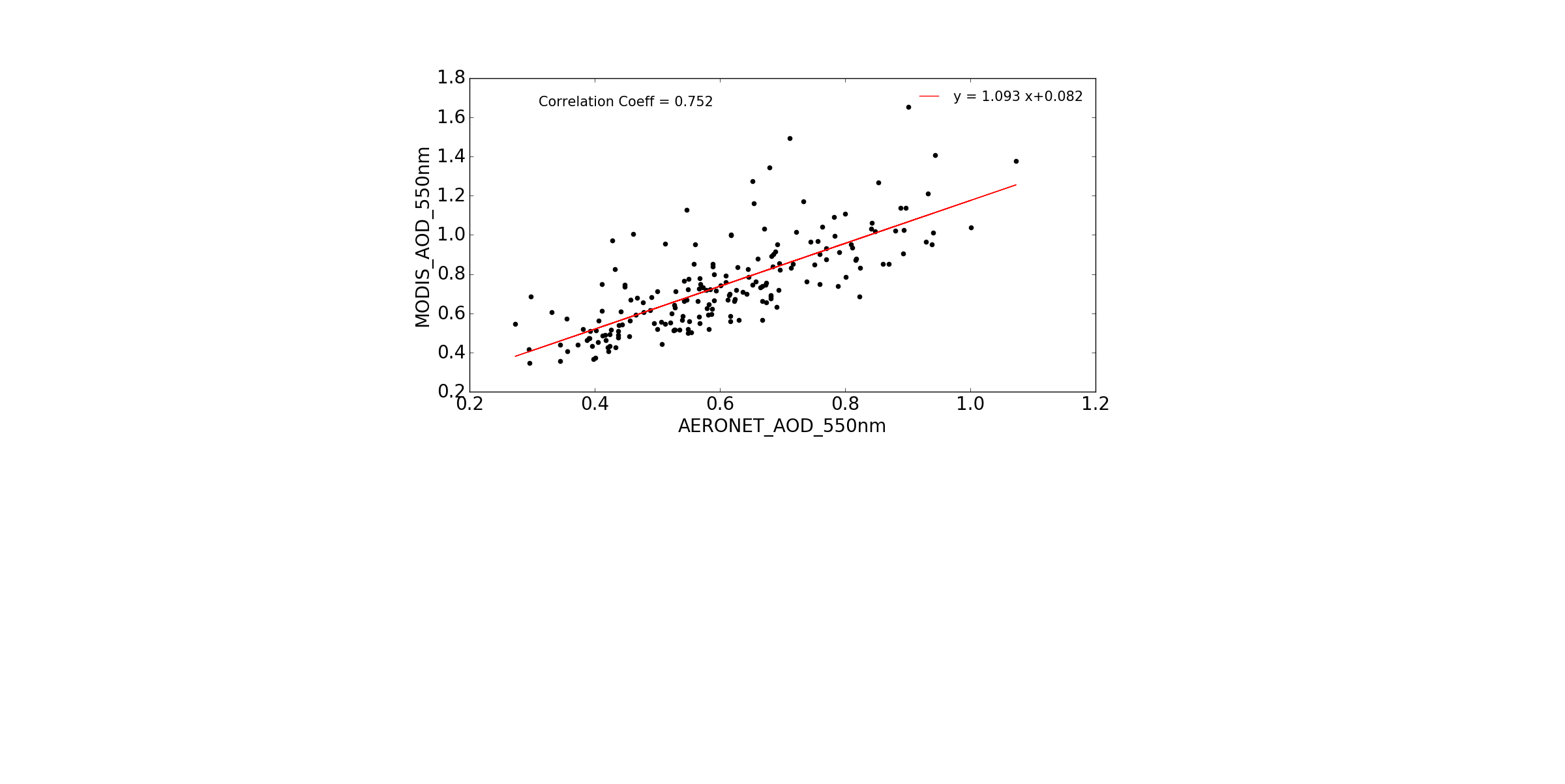


**Figure S10.** Comparison between monthly mean AOD obtained from MODIS and AERONET observations over Kanpur (26.5°N, 80.2°E) region obtained during 2000-2018. MODIS observations within 1° spatial resolution over Kanpur station is used.


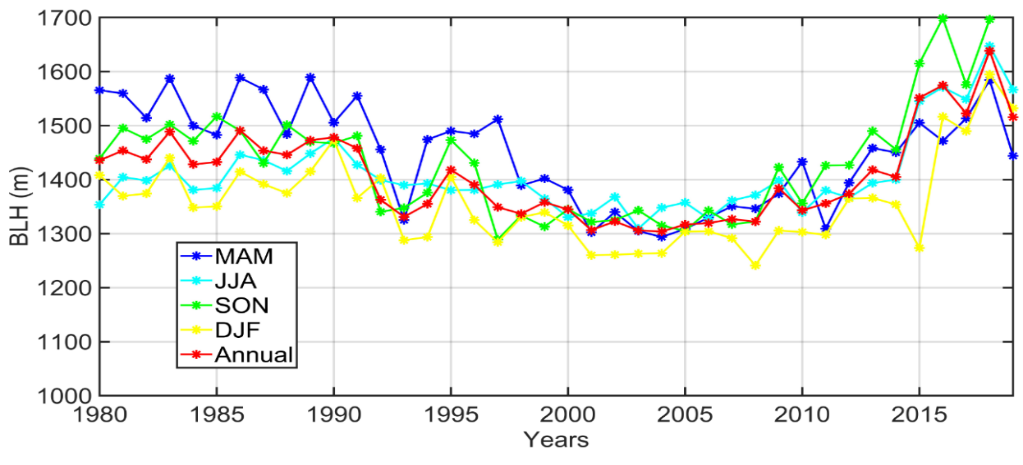

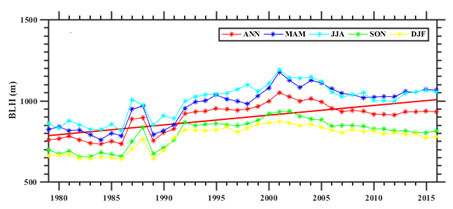


(a)

(b)

**Figure S11**. Time series of annual mean (in red) and seasonal mean (MAM in blue, JJA in cyan, SON in green, DJF in yellow) boundary layer height for the period 1980 to 2019 over India from 31 radiosonde stations (a) and over China from 1979-2016 (Guo et al., 2019, Figure S3) (b).
